# LncRNA HOXA-AS2 Promotes Temozolomide Resistance in Glioblastoma by Regulated miR-302a-3p/IGF1 Axis

**DOI:** 10.1155/2022/3941952

**Published:** 2022-11-21

**Authors:** Ligang Lin, Da Lin, Lingjiang Jin, Junyou Wang, Zheng Lin, Shuai Zhang, Gaojun Lin

**Affiliations:** ^1^Neurosurgery, The First People's Hospital of Wenling, Wenling Hospital Affiliated to Wenzhou Medical University, Wenling, Zhejiang, China; ^2^Department of Critical Care Medicine, The First People's Hospital of Wenling, Wenling Hospital Affiliated to Wenzhou Medical University, Wenling, Zhejiang, China

## Abstract

**Background:**

Glioblastoma (GBM) is a highly prevalent brain tumor characterized by high rates of morbidity, recurrence, and mortality. While temozolomide (TMZ) is commonly used as a first-line treatment for this cancer, the emergence of TMZ resistance limits its utility. The long noncoding RNA HOXA-AS2 reportedly drives GBM progression, but whether it can influence therapeutic resistance to TMZ has yet to be established.

**Methods:**

HOXA-AS2 expression was analyzed in TMZ-resistant and sensitive GBM tissue samples and cell lines by qPCR. A siRNA-based approach was used to knock down HOXA-AS2 in GBM cells, after which TMZ resistance was tested. Bioinformatics approaches were used to predict miRNA binding targets of HOXA-AS2, after which a series of luciferase reporter assay and rescue experiments with appropriate miRNA inhibitor/mimic constructs were performed to validate these predictions and to clarify the ability of HOXA-AS2 to regulate chemoresistant activity.

**Results:**

TMZ-resistant GBM patients and cell lines exhibited increased HOXA-AS2 expression that was correlated with worse overall survival. Knocking down HOXA-AS2 increased the sensitivity of resistant GBM cells to TMZ. miR-302a-3p was identified as a HOXA-AS2 target confirmed through luciferase reporter assays and rescue experiments, and IGF1 was further identified as a confirmed miR-302a-3p target. In addition, HOXA-AS2 knockdown resulted in a corresponding drop in IGF1 expression consistent with indirect regulation mediated by miR-302a-3p.

**Conclusion:**

In summary, these results highlight the role of HOXA-AS2 as a driver of TMZ resistance in GBM through its ability to regulate the miR-302a-3p/IGF1 signaling axis, highlighting this pathway as a promising target for the diagnosis, therapeutic sensitization, and/or treatment of affected patients.

## 1. Introduction

Glioblastoma (GBM) is the most common form of primary intracranial malignancy, accounting for 40–50% of all brain tumor cases [1]. GBM patients exhibit symptoms associated with intracranial compression and pressure and exhibit low cure rates together with high rates of morbidity, recurrence, and death [2–4]. The oral alkylating agent temozolomide (TMZ) exhibits 100% bioavailability as is able to readily transit across the blood-brain barrier without causing significant treatment-related side effects, leading to its frequent utilization as a first-line chemotherapeutic drug in GBM patients following surgical tumor removal [5]. However, these patients often develop resistance to TMZ during the later stages of treatment, markedly limiting its utility and contributing to high rates of failed treatment [6]. The etiology of TMZ resistance is complex and multifactorial, highlighting the need for further studies of these chemoresistant processes in an effort to better provide GBM patients with effective pharmacological interventions.

Long noncoding RNAs (lncRNAs) lack coding potential despite being >200 nucleotides in length, yet nonetheless serve as important regulators of diverse biological processes at the transcriptional, posttranscriptional, and epigenetic levels [7]. Mechanistically, these lncRNAs can control the onset and progression of tumors through epigenetic modification, chromatin recruitment, and alternative splicing and represent promising diagnostic and therapeutic targets in many cancers [8]. Specific lncRNAs have been reported to be associated with the development of chemoresistant characteristics in breast cancer [9] and cutaneous squamous cell carcinoma [10]. There is also evidence for the ability of certain lncRNAs to shape tumor cell resistance to TMZ [11, 12]. Efforts to target these lncRNAs may thus represent a novel TMZ sensitization strategy. In prior reports, the lncRNA HOXA-AS2 was found to target the miR-885-5p/RBBP4 axis to influence GBM malignancy [13]. However, the role this lncRNA plays in the context of TMZ chemoresistance or the underlying mechanisms governing such activity remain poorly understood.

Here, HOXA-AS2 was found to target miR-302a-3p and thereby indirectly promote IGF1 expression and signaling activity in GBM cells, thereby enhancing resistance to TMZ, as confirmed through analyses of GBM tissue samples and cell lines. Accordingly, these findings suggest that targeting HOXA-AS2 may represent an effective means of overcoming GBM resistance to TMZ treatment.

## 2. Materials and Methods

### 2.1. Patients Samples

In total, 264 glioma tissue samples were collected from individuals at the First People's Hospital of Wenling that had undergone surgery and chemotherapy. The Ethics Committee of the First People's Hospital of Wenling approved this study, and all patients provided written informed consent.

### 2.2. Cell Culture

The U87 and U251 human GBM cell lines were obtained from the Cell Bank of the Chinese Academy of Sciences (Shanghai, China) with TMZ-resistant substrains of these cell lines (U87TR and U251TR) having been established by our laboratory. Cells were grown in DMEM (Invitrogen) supplemented with 10% FBS (Hyclone, UT, USA) and penicillin/streptomycin in a 37°C 5% CO_2_ humidified incubator (Thermo Scientific, MA, USA). TMZ resistance was maintained by alternating the culture of U87TR and U251TR cells in media supplemented with TMZ (Sigma, CA, USA).

### 2.3. Viability and TMZ Chemosensitivity Analyses

After addition to 96-well plates, GBM cells were treated for 48 h with TMZ. Media was then exchanged for media supplemented with 10% CCK-8 solution for 2 h (Dojindo, Kumamoto, Japan), after which absorbance at 450 nm was assessed via ultra multifunctional microplate reader (Tecan, Switzerland), with IC50 values being determined as a measurement of cell sensitivity to TMZ treatment.

### 2.4. RNA Immunoprecipitation

A magna RIP RNA-binding protein immunoprecipitation kit (Bersinbio, Guangzhou, China) was used based on provided directions to complete all RNA immunoprecipitation (RIP) assays. Briefly, RIP lysis buffer was used to lyse cells which were then separated into equal volumes and incubated overnight with 5 *μ*g of human antiargonaute2 (AGO2) (Millipore, MA, USA) or control IgG (Millipore) at 4°C with constant shaking. RNA was then purified for qPCR analysis. The purified RNA was used to detect the expression levels of the genes of interest by RT-qPCR.

### 2.5. qPCR

Trizol (Takara Bio, Shiga, Japan) was used based on provided directions to extract total RNA from appropriate samples, with RNA yield and quality being determined through measurements of absorbance values at 260 and 280 nm. The M-MLV reverse transcriptase (Promega, WI, USA) was used to prepare cDNA for HOXA-AS2 analyses, whereas the prime script TM RT reagent kit (Takara Bio, Shiga, Japan) was used to prepare cDNA for analyses of mRNA expression. All qPCR reactions were performed with the SYBR GREEN PCR master mix (Takara Bio, Shiga, Japan) and a 7500 fast real-time PCR instrument (Applied Biosystem, CA, USA).

### 2.6. Statistical Analysis

Data are means ± SD from triplicate analyses and were compared using Student's *t*-tests or one-way ANOVAs with Tukey's posthoc test. Mann–Whitney *U*-tests were used to compare data for tumor samples. The relationship between HOXA-AS2 expression and patient survival was examined using Kaplan–Meier curves. All analyses were conducted using SPSS 19.0 (SPSS Inc, IL, USA) and GraphPad Prism 7.0 (GraphPad Software, Inc, CA, USA).

## 3. Results

### 3.1. TMZ-Resistant GBM Tissues and Cells Exhibit HOXA-AS2 Upregulation

**Figure 1:**
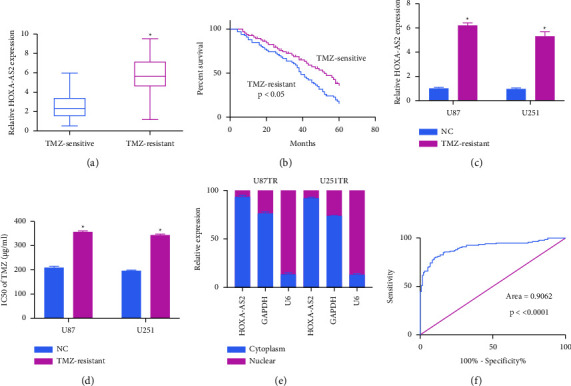
TMZ-resistant GBM patient tissues and cells exhibit HOXA-AS2 upregulation. (a) Levels of HOXA-AS2 were assessed in TMZ-resistant (*n* = 132) and sensitive (*n* = 132) GBM patient tissues. (b) The survival of TMZ-resistant (*n* = 132) and sensitive (*n* = 132) GBM patients was assessed with Kaplan–Meier curves. (c) HOXA-AS2 levels were detected in TMZ-resistant GBM (GBMTR) cells. (d) GBMTR cell viability and corresponding IC50 values were computed through CCK-8 assays. (e) Nuclear and cytoplasmic HOXA-AS2 levels were detected in GBMTR cells. (f) HOXA-AS2 ROC curve analyses. ^*∗*^*P*  <  0.05.

When comparing TMZ-sensitive and TMZ-resistant GBM patient tissue samples, the expression of HOXA-AS2 was increased in the context of TMZ resistance (Figure 1(a)), with such resistance also being correlated with significantly decreased patient's overall survival (Figure 1(b)). To validate these findings, GBM cell lines were next interrogated, revealing higher HOXA-AS2 expression levels in TMZ-resistant cell lines (U87TR and U251TR) relative to corresponding parental cells (Figure 1(c)). As expected, these U87TR and U251TR cell lines exhibited superior TMZ resistance (Figure 1(d)), and more detailed analyses revealed HOXA-AS2 to be preferentially enriched in the cytosol relative to the nuclear fraction (Figure 1(e)). Receiver operating characteristic (ROC) curves suggested that HOXA-AS2 expression may offer value as a biomarker capable of predicting GBM patient resistance to TMZ treatment (Figure 1(f)).

### 3.2. Knocking down HOXA-AS2 Suppresses the Ability of GBM Cells to Resist TMZ in Vitro

**Figure 2:**
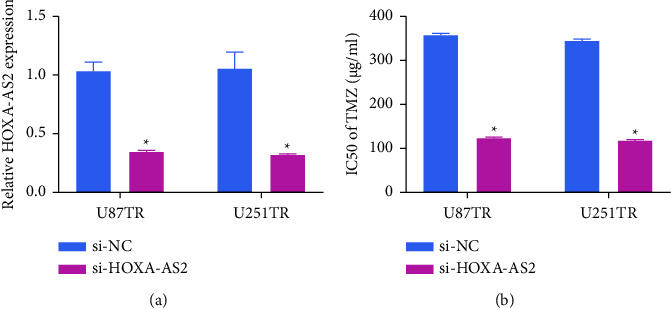
Knocking down HOXA-AS2 suppresses TMZ-resistant GBM cell chemoresistance. (a) HOXA-AS2 levels were detected in TMZ-resistant GBM (GBMTR) cells via qPCR. (b) The viability of GBMTR cells and corresponding IC50 values were computed following a series of TMZ treatments. ^*∗*^*P*  <  0.05.

Next, a knockdown approach was used to suppress HOXA-AS2 expression in U87TR and U251TR, as confirmed via qPCR (Figure 2(a)). Subsequent viability and drug sensitivity analyses indicated that TMZ IC50 values were significantly reduced following treatment with a HOXA-AS2-specific siRNA rin both tested TMZ-resistant cell lines as compared to control si-NC transfection (Figure 2(b)). Accordingly, these data confirmed the ability of HOXA-AS2 silencing to suppress GBM cell resistance to TMZ *in vitro.*

### 3.3. HOXA-AS2 targets miR-302a-3p to Enhance GBM Cell TMZ Resistance

**Figure 3:**
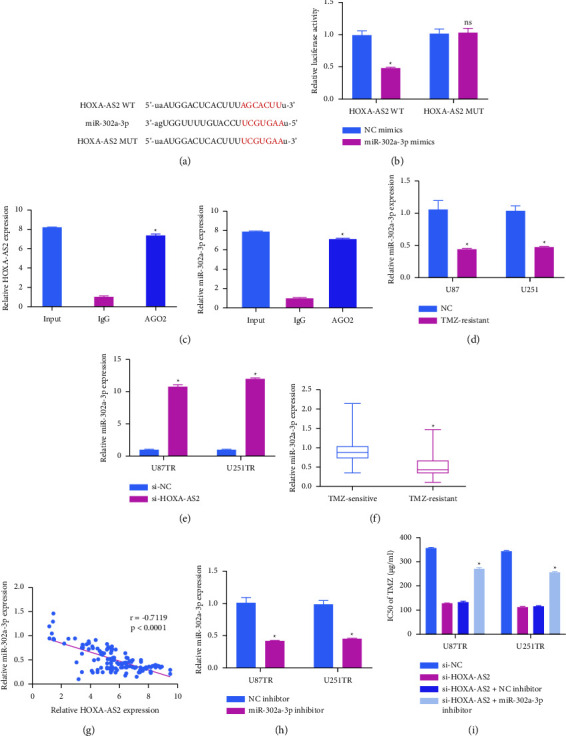
Knockdown of HOXA-AS2 results in miR-302a-3p upregulation and GBM cell sensitization to TMZ. (a) HOXA-AS2 was identified as a putative ceRNA for miR-302a-3p. (b) Luciferase reporter assays were conducted by transfecting HEK293T cells using miR-302a-3p and WT or mutated (MUT) HOXA-AS2 reporter vectors. (c) HEK293T cells were utilized to conduct an anti-AGO2 RIP assay, after which miR-302a-3p and HOXA-AS2 levels in precipitated samples were detected via qPCR. (d) miR-302a-3p levels were detected in GBMTR cells. (e) Changes in miR-302a-3p expression following HOXA-AS2 knockdown were examined in GBMTR cells via qPCR. (f) Levels of miR-302a-3p were assessed in TMZ-resistant (*n* = 132) and sensitive (*n* = 132) GBM patient tissues. (g) HOXA-AS2 and miR-302a-3p levels in TMZ-resistant GBM tissue samples were negatively correlated. (h) Following miR-302a-3p inhibitor or control construct transfection, levels of this miRNA were detected via qPCR in GBMTR cells. (i) Following exposure to the indicated TMZ doses, GBMTR cell viability and corresponding IC50 values were detected via CCK-8 assay. ^*∗*^*P*  <  0.05.

Predictive bioinformatics analyses suggested miR-302a-3p to be a putative HOXA-AS2 target miRNA (Figure 3(a)). Consistently, miR-302a-3p overexpression in a luciferase reporter assay system was sufficient to suppress the activity of the wild-type HOXA-AS2 reporter, whereas the mutated version of this reporter was unaffected (Figure 3(b)). Consistently, RIP assays confirmed the identity of miR-302a-3p as a HOXA-AS2 target (Figure 3(c)) and TMZ-resistant GBM cells exhibited lower miR-302a-3p expression (Figure 3(d)), while such expression was enhanced following the silencing of HOXA-AS2 (Figure 3(e)). In addition, relative to tissue samples from TMZ-sensitive GBM patients, those from individuals resistant to TMZ exhibited lower levels of miR-302a-3p (Figure 3(f)). Moreover, HOXA-AS2 and miR-302a-3p expression levels were negatively correlated in TMZ-resistant GBM patient tissue samples (Figure 3(g)). When miR-302a-3p expression was knocked down with an inhibitor constructed in TMZ-resistant cells (Figure 3(h)), this reversed the effect of HOXA-AS2 knockdown on the TMZ sensitivity of these cells (Figure 3(i)). Accordingly, these results confirmed that the ability of HOXA-AS2 to enhance the resistance of GBM cells to TMZ treatment was related to the ability of this lncRNA to interact with miR-302a-3p.

### 3.4. miR-302a-3p Targets IGF1 to Reduce GBM Cell Resistance to TMZ

**Figure 4:**
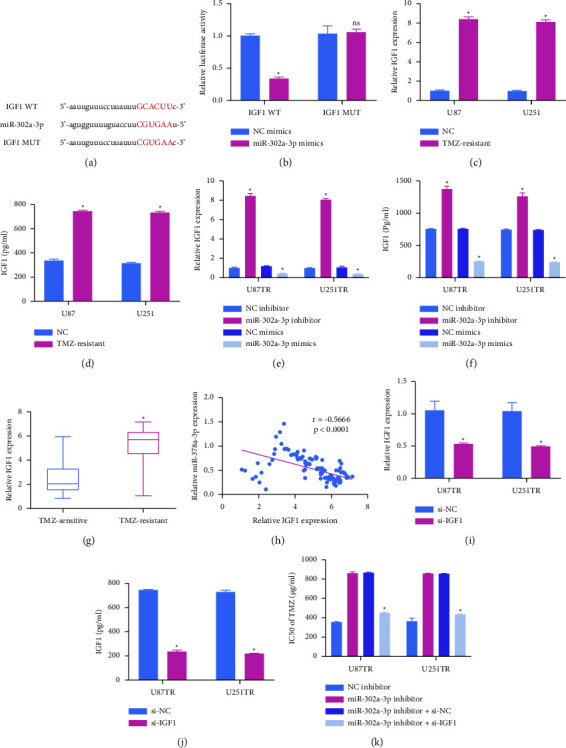
miR-302a-3p targets IGF1 and thereby reduces GBM cell resistance to TMZ. (a) IGF1 was identified as a putative miR-302a-3p target gene. (b) HEK293T cells were used to perform luciferase assays in which cells had been cotransfected with miR-302a-3p and WT or mutated (MUT) IGF1 3'-UTR reporter constructs. (c, d) IGF1 levels were detected in GBMTR cells via qPCR (c) and ELISA (d). (e, f) IGF1 levels in GBMTR cells were analyzed via qPCR (e) or ELISA (f) following transfection with miR-302a-3p inhibitor, mimic, or NC control constructs. (g) Levels of IGF1 were assessed in TMZ-resistant (*n* = 132) and sensitive (*n* = 132) GBM patient tissues. (h) IGF1 levels were negatively correlated with miR-302a-3p levels in tissue samples from GBM patients resistant to TMZ. (i, j) Both qPCR (i) and ELISA (j) approaches were employed to measure IGF1 levels in GBMTR cells following IGF1-specific or control siRNA transfection. (k) GBMTR cell viability was measured via CCK-8 assay after treatment with the indicated TMZ doses, and IC50 values were calculated. ^*∗*^*P*  <  0.05.

The TargetScan database identified IGF1 as a putative miR-302a-3p target (Figure 4(a)). Consistently, the cotransfection of 293T cells with miR-302a-3p mimic constructs and luciferase reporter vectors harboring WT or mutated versions of the 3'-UTR of IGF1 revealed that miR-302a-3p was only able to effectively bind the WT version of this sequence to modulate luciferase activity (Figure 4(b)). Accordingly, TMZ-resistant cells were found to exhibit increased IGF1 expression as detected via qPCR and ELISA (Figures 4(c) and 4(d)), while miR-302a-3p inhibitor transfection enhanced IGF1 levels in these TMZ-resistant cells, and miR-302a-3p mimic transfection had the opposite effect (Figures 4(e)–4(f)). Marked upregulation of IGF1 was evident in tumor tissues from TMZ-resistant patients with GBM (Figure 4(g)), with a corresponding negative correlation between IGF1 and miR-302a-3p in this cohort of patient samples (Figure 4(h)). Further analysis of the ability of this miR-302a-3p/IGF1 regulatory axis to control TMZ resistance revealed that knocking down IGF1 in TMZ-resistant GBM cells (Figures 4(i) and 4(j)) reversed the effects of miR-302a-3p inhibitor treatment on the TMZ IC50 value for these cells (Figure 4(k)). These findings thus confirmed the ability of miR-302a-3p to target IGF1 and to thereby overcome GBM cell chemoresistance.

### 3.5. HOXA-AS2 Promotes IGF1 Upregulation Via Sequestering miR-302a-3p

**Figure 5:**
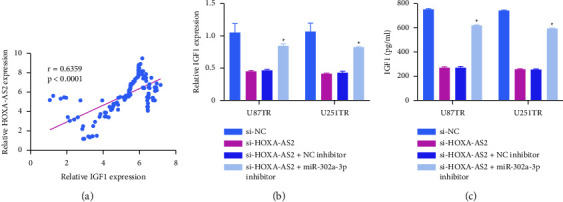
HOXA-AS2 sequesters miR-302a-3p and serves as a positive regulator of IGF1. (a) Correlations between the expression of HOXA-AS2 and IGF1 in tissues from TMZ-resistant GBM patients. (b, c) levels of IGF1 were measured in GBMTR cells via qPCR (b) and ELISA (c). ^*∗*^*P*  <  0.05.

Given the above data demonstrating the ability of HOXA-AS2 to bind miR-302a-3p and the ability of this miRNA to in turn suppress IGF1 expression, a final set of experiments was performed to examine the ability of HOXA-AS2 to indirectly regulate IGF1 by serving as a competing endogenous RNA (ceRNA) specific for miR-302a-3p. GBM patient tissues exhibited positive correlations between HOXA-AS2 and IGF1 levels (Figure 5(a)), and the knockdown of HOXA-AS2 in TMZ-resistant cell lines resulted in a corresponding drop in IGF1 expression at the mRNA and protein levels, while this was reversed by miR-302a-3p inhibitor treatment (Figures 5(b) and 5(c)). Overall, these results were consistent with the ability of HOXA-AS2 to bind miR-302a-3p and thereby indirectly modulate the expression of IGF1.

## 4. Discussion

Resistance to TMZ represents a major roadblock to the effective treatment of GBM, highlighting a critical need for further therapeutic innovation. Here, the association between HOXA-AS2 expression and such chemoresistance was analyzed in GBM patients and cells. The knockdown of HOXA-AS2 *in vitro* ultimately revealed that it functions to promote resistance to TMZ in GBM cells along the miR-302a-3p/IGF1 axis. The upregulation of HOXA-AS2 was evident in both TMZ-resistant GBM patient samples and cells. These data align with prior evidence that HOXA-AS2 can serve as a regulator of GBM progression [13], and with other research suggesting that lncRNAs can act as critical modulators of chemoresistance in head and neck squamous cell carcinoma [14], colorectal cancer [15], and nonsmall cell lung cancer [16]. These findings thus suggest that HOXA-AS2 may represent a novel mediator of resistance to TMZ in GBM.

While initially disregarded as a form of transcriptional noise, more recent work has characterized the importance of lncRNAs as physiological regulators in diverse contexts. Mechanistically these noncoding RNAs can act as ceRNAs for particular miRNAs, can interact with specific proteins, and can regulate transcriptional activity and splicing [17–19]. The ceRNA mechanism of action has been a focus of key research interest in recent years, offering a pathway whereby these lncRNAs can influence therapeutic responsiveness. Lnc-TALC, for example, was found to bind to miR-20b-3p in a competitive manner and thereby drive the expression of O6-methylguanine-DNA methyltransferase through c-Met pathway regulation [20], with the NEAT1 lncRNA that can regulate the let-7g-5p/MAP3K1 pathway to control the malignancy of glioblastoma stem cells and their ability to resist TMZ treatment [11].

Here, HOXA-AS2 was identified as an inducer of the onset of TMZ resistance owing to its ability to competitively bind miR-302a-3p and thereby upregulate IGF1. These findings are consistent with prior evidence for the ability of miR-302a-3p to serve as an oncogenic mediator in testicular germ cell tumors [21]. However, this miRNA has not previously been reported to act as a regulator of chemoresistance. IGF1 has been shown to enhance melanoma cell resistance to cisplatin [22], in line with the prior data. Indeed, TMZ-resistant GBM patient tissue samples and cells exhibited increased IGF1 expression, and the knockdown of HOXA-AS2 or the upregulation of miR-302a-3p was sufficient to reduce IGF1 levels in TMZ-resistant cells. Overall, these findings thus support the existence of a HOXA-AS2/miR-302a-3p/IGF1 regulatory axis that controls the ability of GBM cells to resist TMZ. The mechanisms whereby IGF1 controls such resistance and the clinical relevance of this regulatory pathway, however, will necessitate further study and experimental validation.

In summary, the present findings indicate that HOXA-AS2 overexpression is evident in GBM patients in the context of TMZ resistance, with the upregulation of this lncRNA representing an important biomarker associated with poor prognostic outcomes. From a mechanistic perspective, knocking down HOXA-AS2 was sufficient to alleviate GBM cell resistance to TMZ via the miR-302a-3p/IGF1 axis. Together these data offer new insight regarding chemoresistance in GBM, potentially highlighting novel avenues for future therapeutic intervention.

## Data Availability

The data used to support the findings of this study are available from the corresponding author upon request.
